# Photocatalyzed elaboration of antibody-based bioconjugates

**DOI:** 10.3762/bjoc.21.49

**Published:** 2025-03-18

**Authors:** Marine Le Stum, Eugénie Romero, Gary A Molander

**Affiliations:** 1 Université Paris-Saclay, CEA, INRAE, Département Médicaments et Technologies pour la Santé (DMTS), SCBM, 91191 Gif-Sur-Yvette, France

**Keywords:** antibodies, bioconjugation, bioorthogonality, chemoselectivity, late-stage functionalization, photochemistry

## Abstract

Antibody–drug conjugates (ADCs) represent a promising class of targeted therapeutics, combining the specificity of antibodies with the potency of cytotoxic drugs to enhance therapeutic efficacy while minimizing off-target effects. The development of new chemical methods for bioconjugation is essential to generate ADCs and to optimize their stability, efficacy, and safety. Traditional conjugation methods often face challenges related to site-selectivity and heterogeneous product mixtures, highlighting the need to develop new, innovative chemical strategies. Photoredox chemistry emerges as a powerful tool in this context, enabling precise, mild, and selective modifications of peptides and proteins. By harnessing light to drive chemical transformations, photoredox techniques can facilitate the synthesis of antibody bioconjugates. This perspective will discuss the drive to develop and empower photoredox methods applied to antibody functionalization.

## Introduction

Antibodies represent increasingly important tools in several groundbreaking approaches to medical innovation, including basic biomedical research and therapy. One of the most critical requirements for the application of antibodies in disease detection and treatment is that they are adorned with functional units (e.g., fluorophores, radionuclides, toxic payloads) that must be efficiently and selectively installed through compatible synthetic methods.

Dozens of ADCs have been approved for clinical uses, and to date all are designed in the context of cancer therapy [1], which combines the precision targeting of monoclonal antibodies (mAbs) with the therapeutic effects of cytotoxic drugs [2]. The ADCs are thus designed to deliver potent cytotoxic agents selectively and directly to cancer cells while minimizing damage to healthy tissues. Notably, ADCs have started to enter clinical trials for non-oncology applications as well [3].

The importance and value of ADCs are several fold:

- Precise targeting: ADCs specifically recognize their target cells because of their antibody component. This minimizes collateral damage to healthy tissues, reducing side effects compared to traditional chemotherapy.
- Enhanced potency: By delivering cytotoxic payloads directly to tumor or other target cells, ADCs achieve higher drug concentrations at the site of action. This potency enhances therapeutic efficacy.
- Treatment of refractory diseases: ADCs have shown remarkable success in treatment of highly refractory diseases. Their ability to overcome resistance makes them valuable options for patients who previously had limited treatment choices.

**Figure 1:**
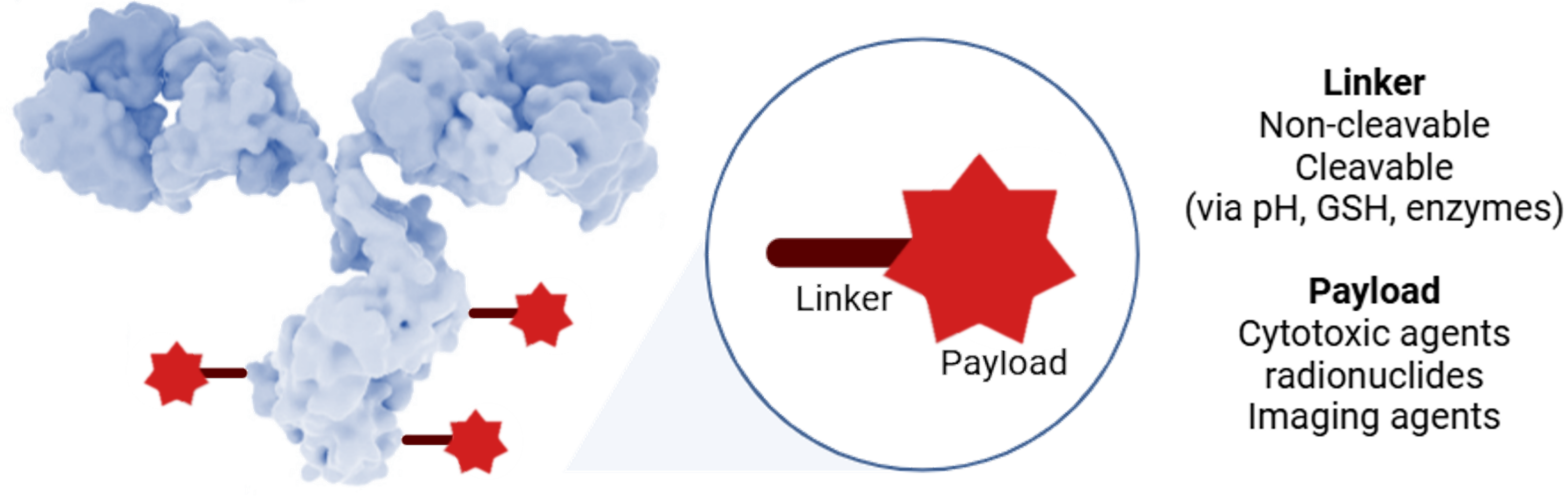
Representation of an antibody–drug conjugate. The antibody shown in this figure is from https://www.gettyimages.de/detail/illustration/monoclonal-antibody-igg2a-lizenfreie-illustration/585105259?searchscope=image%2Cfilm&adppopup=true; ALFRED PASIEKA/SCIENCE PHOTO LIBRARY, No. 585105259. This content is not subject to CC BY 4.0.

ADCs consist of three main components (Figure 1): (1) Monoclonal antibody (mAb): The antibody specifically recognizes and binds to surface antigens present on tumor or other targeted cells. (2) Linker: the linker connects the antibody to the payload. The nature of the moiety linking the drug/radiolabel/imaging agent to the antibody plays a crucial role in the pharmacokinetic properties [4–5], therapeutic index, selectivity, and overall success of the ADC. The linker will ideally be stable in plasma for an extended period before the intended target can be reached, and after internalization, linkers play a key role in the drug-releasing event. Importantly, some exceptions exist. For example, in trodelvy, used in a treatment for patients with triple-negative breast cancer, the linker may release the drug prior to internalization. (3) Payload: The payload may be a subunit used in cellular tracking, imaging, or most commonly toxic drug therapeutics. The overall goal of ADCs is to deliver the payload directly to target cells via the antibody without affecting normal, healthy tissue.

Importantly, the use of antibodies in modern medicine is not restricted solely to ADCs and cancer therapy. For example, mABs now find routine use in the context of radionuclide (PET) imaging agents, informing therapeutic decision-making [6–7]. Antibody–oligonucleotide conjugates, antibody–enzyme conjugates, antibody–polymer conjugates, antibody–nanomaterial conjugates, antibody–catalyst conjugates, and antibodies involved in protein degradation also play critical roles in biomedical research and therapies [2].

In whatever capacity, the use of antibodies applied to medicine is critically dependent on the ability to anchor them to operative payloads in a highly precise manner [8–10]. Many researchers are tackling the challenge of new strategies for chemoselective synthetic modifications of biomolecules, including antibodies [11]. In the case of ADCs, conjugation of the linker/payload to the antibody must not drastically alter the pharmacokinetics or physicochemical properties of the antibody [12–13]. Typically, zero to eight payloads are attached to the antibody. Heterogeneous ADCs may thus be a mix of both unconjugated and “overloaded” antibodies. Unconjugated antibodies compete with antibodies containing payloads for binding, which can diminish the effectiveness of the antibody-payload materials. On the other hand, excessive loading of the antibody can lead to antibody aggregation, increased toxicity, decreased stability, and/or a shorter ADC half-life [14]. Optimization of the drug/antibody ratio (DAR) and payload distribution/location thus becomes significant for ideal ADC design.

Given the complexity of biological macromolecules, there are inherent limitations in terms of the types of reactions that can be used to modify them. Reactions must be carried out at or near ambient temperatures and near-neutral pH. Aqueous media is often required to solubilize the substrates and/or to prevent denaturation, and reactions are normally carried out under very dilute (micromolar) conditions. As importantly, the highly functionalized nature of polypeptides/proteins demands exquisite selectivity to target specific sites on the macromolecule of interest. In addition to the requisite features of the chemical methods outlined above, there are key requirements for synthetic methods used in ADCs, which include high site-selectivity and controlled stoichiometry (DAR). Additionally, the methods should be synthetically reliable, easy, and rapid to carry out, and they must tolerate diverse functional groups on the payloads/linker [15–16]. These factors conspire to set a very high bar for those processes that are amenable to covalent bioconjugation.

Effective bioconjugation has been dramatically facilitated by the development of robust bioorthogonal reactions, which has revolutionized the field of chemical biology [17]. A bioorthogonal reactive group can be introduced into proteins via direct chemical coupling on specific native amino acids, by enzymatic coupling, or by bioengineering for the incorporation of non-canonical amino acids [18–19]. In fact, incorporating non-natural amino acids significantly broadens the scope of reactions that can be used in bioconjugation. As an example, click chemistry comprises one particularly useful and important tool in which azides and other dipolar species engage with reactive alkenes and alkynes on non-canonical amino acids [20]. Transition-metal-mediated processes, including metathesis reactions, aryl cross-coupling reactions, and conjugate addition reactions with dehydroalanine derivatives round out the most predominant reactions used on non-natural amino acids. However, even though bioengineering allows the incorporation of non-canonical amino acids with astounding effectiveness and near-complete selectivity, this technology is exceedingly expensive and time-consuming, and the expertise required to carry out these transformations is constrained to a very limited number of laboratories, particularly in the field of antibodies.

Given the challenges of bioengineering antibodies with non-canonical amino acids, the direct and chemoselective modification of native antibodies is most attractive, albeit not without serious obstacles. Among native amino acids, attachment to lysine (Lys) [21] and cysteine (Cys) are the most common. Antibodies typically contain as many as 90 lysine units, many of which are at highly solvent-accessible sites [22–23]. Conjugation to Lys is thus rarely controlled and leads to a wide range of DARs that can destabilize the antibody and furthermore greatly alter the pharmacokinetics of the ADC (vide infra). Additionally, undesirable Cys and Tyr modifications may compete in some instances with the Lys conjugation.

Cysteine amino acids are less prevalent and typically more evenly distributed in antibodies than lysine but are often tied up in disulfide bridges that must be reduced prior to conjugation [24–25]. Such reductions must thus be carefully controlled to allow conjugation while ensuring the overall structural integrity of the antibody. The free sulfhydryls can be conjugated by several means, including alkylation with α-halo carbonyls (in which case Lys may compete), and Michael additions (e.g., to maleimide, which is reversible and therefore may lead to incomplete conversion). The Michael adducts also present chemical instability in plasma and additionally generate an undesirable new stereocenter [26–27].

Few methods have been developed for the functionalization of tyrosine (Tyr) and tryptophan (Trp). With a low abundance (≈3%) in proteins, Tyr modifications are widely recognized for their ability to profoundly impact protein properties and function. Their chemical modification has been developed using iminoxyl radicals [28]. Although numerous methods exist for the functionalization of Trp in proteins, their application in the elaboration of ADCs is limited [29].

**Figure 2:**
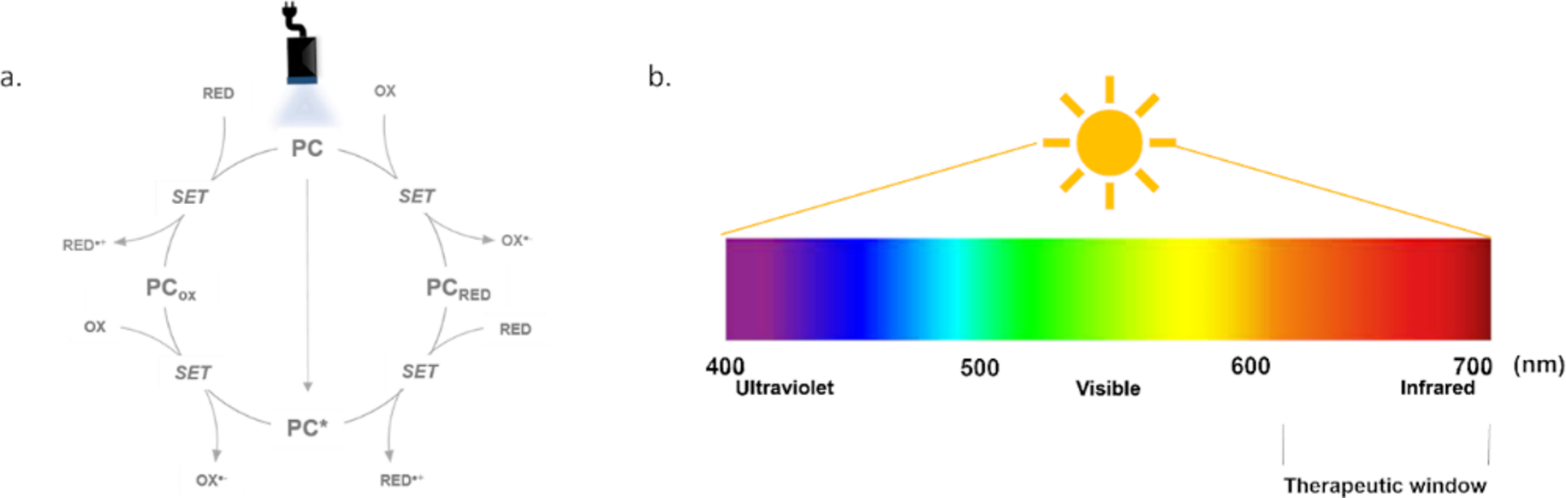
a. Photoredox catalytic cycles; b. absorption spectrum of photosensitizers. Therapeutic window indicates the most appropriate wavelength range to apply irradiation for biological applications.

Given the many challenges in antibody modification as outlined above, it is perhaps of no surprise that the recent renaissance of synthetic chemistry based on novel photochemical methods has been applied to their functionalization [30]. In the context of photochemistry applied to bioconjugation, photochemical affinity labelling (PAL) is a technique that has been used to create selective chemical reactions, often for bioconjugation, by utilizing photoaffinity reagents. These reagents are typically photoreactive molecules that can be activated by light to form covalent bonds with nearby molecules [31–33]. PAL involves using a light-activated group (often a photo-crosslinker) that can form covalent bonds when exposed to UV or visible light. These photoaffinity tags or crosslinkers are designed to bind to specific biomolecules, such as proteins, nucleic acids, or lipids, in a highly selective manner. The light irradiation triggers a chemical reaction, such as a bond formation, which allows the conjugation of two molecules. PAL is commonly used in targeted bioconjugation when the timing and location of the conjugation need to be controlled. A typical example is using azido groups or alkyne groups in conjunction with light to initiate a covalent bond between two different molecules, such as a drug and a targeting moiety. This technique has been shown to be useful in applications such as cell labeling, protein–protein interactions, and photoradiosynthesis of bioconjugates, but the most important challenge remains the lack of specificity to target one amino acid, and thus to have a better control of the selectivity and the DAR, leading to more homogeneous ADCs. In this specific context, photocatalysis (Figure 2) enables site-specific bioconjugation by generating reactive intermediates (such as radicals or electron-deficient species) that can selectively react under the agency of low-energy visible light with specific functional groups on biomolecules. This allows precise control over the conjugation process, enabling targeted modifications without affecting non-reactive sites.

Although previous reviews have discussed the more traditional synthetic methods applied to ADCs [34–36], the discussion below focuses on photocatalytic approaches that have been used to date to elaborate antibodies, providing insight into those methods that aim to revolutionize approaches to cancer treatment and other medical applications via the use of synthetically functionalized antibodies.

## Perspective

In this perspective, how light-driven chemistry can enhance the development of innovative methods for accessing antibody–drug conjugates (ADCs) will be outlined. A brief introduction to photoredox chemistry as it relates to bioconjugation in proteins is followed by a summary of the limited photoredox approaches reported for antibodies. Finally, the potential benefits and cautionary details these chemical strategies possess for creating ADCs with well-defined DAR and enhanced selectivity are discussed.

### Photocatalytic modification of proteins

**Figure 3:**
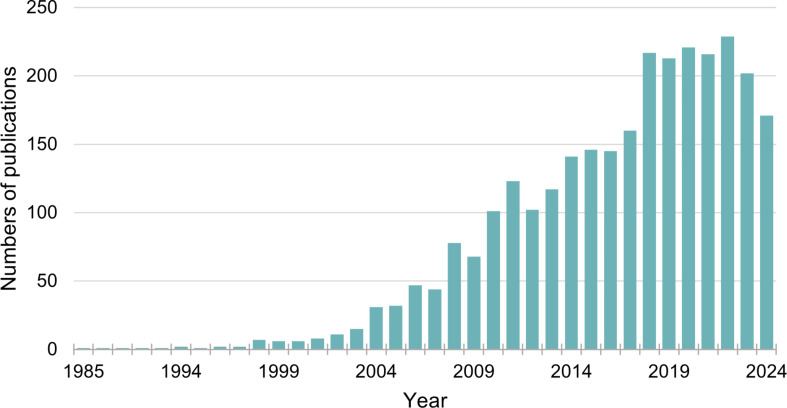
Graph representing the average number of publications focusing on photoredox chemistry applied to protein-based bioconjugation (source: SciFinder).

Photoredox chemistry has emerged as a transformative approach in the modification of proteins, enabling researchers to achieve selective and efficient conjugation under mild conditions [37]. By utilizing visible light and transition-metal catalysts, this technique allows the generation of reactive intermediates that can facilitate various modifications, including labeling, crosslinking, and the creation of protein–drug conjugates. The incorporation of photoredox strategies has facilitated the synthesis of complex protein architectures, enabling precise control over conjugation sites and degrees of modification. The number of publications reporting photoredox bioconjugation applied to proteins over the last 20 years is significant (Figure 3).

Recent reviews have highlighted advancements in this field, emphasizing methods that enable site-specific modifications of proteins with minimal disruption to their native structures [38–39].

In 2021, Bottecchia and Noël reported the utility of photoredox catalysis for the functionalization of amino acid side chains, paving the way for tailored modifications in biotherapeutics [40]. More recently, Sato et al. have reviewed photochemical strategies enabled by a range of catalysts, including photoredox catalysts, energy-transfer catalysts, and genetically encoded photocatalysts, highlighting their distinct features, mechanisms, applications, and prospects [41]. This thorough analysis showcased the promising advancements in the chemical modification of proteins.

As this field continues to expand, ongoing research efforts are focusing on optimizing reaction conditions, understanding mechanistic pathways, and exploring new catalysts to broaden the scope of photoredox applications in protein chemistry.

The integration of photoredox chemistry with protein modification has opened new avenues for designing advanced biotherapeutics, including ADCs and targeted delivery systems.

### Photoinduced modification of antibodies

Although the bioconjugation of proteins via photocatalytic pathways is well-documented, the application of this method to the functionalization of antibodies remains largely unexplored. This is primarily because of the structural complexity of antibodies, which exhibit a three-dimensional architecture and numerous potential modification sites, making selective control of functionalization sites challenging. Nonetheless, photocatalytic approaches offer a unique potential to overcome these limitations, particularly by enabling modifications under mild and controlled conditions. Unlike classical methods, such as thiol chemistry [22] or widely used click reactions [33], photocatalysis could provide innovative solutions to produce ADCs, especially in terms of selectivity and the preservation of sensitive biological structures when appropriate redox potentials of photocatalysts are applied to the targeted amino acid. Additional advantages of photoinduced reactions include the ability to perform the reactions rapidly (typically <15 minutes). It was only in the late 2010s that the first publications on the subject emerged. An overview of reported photoredox approaches for the functionalization of antibodies is outlined below.

#### Histidine

**Figure 4:**
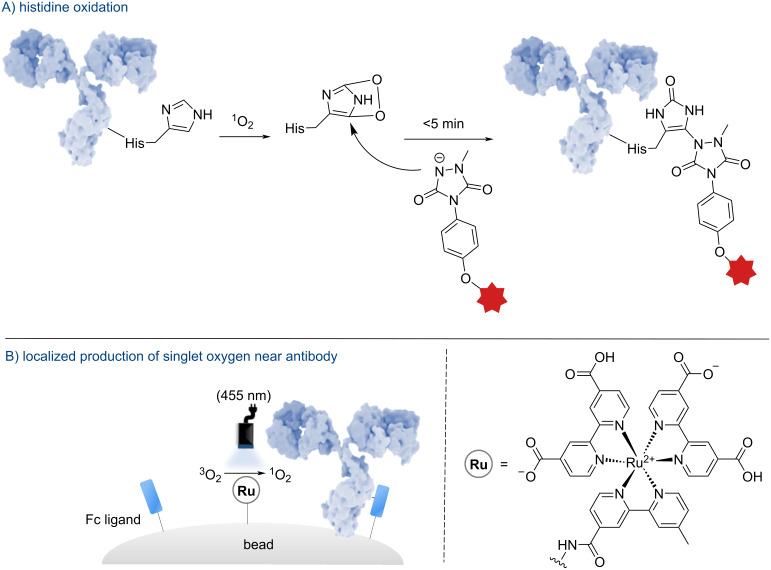
Schematic procedure developed by Sato et al. on histidine photoinduced modification. The antibody shown in this figure is from https://www.gettyimages.de/detail/illustration/monoclonal-antibody-igg2a-lizenfreie-illustration/585105259?searchscope=image%2Cfilm&adppopup=true; ALFRED PASIEKA/SCIENCE PHOTO LIBRARY, No. 585105259. This content is not subject to CC BY 4.0.

In 2021, the group of Sato developed a selective functionalization method for histidine using an umpolung approach based on singlet oxygen [42]. Through energy transfer (EnT) from the ruthenium-based photocatalyst to triplet oxygen, singlet oxygen is produced in a targeted manner, which oxidizes histidine to an endoperoxide, significantly increasing its reactivity toward nucleophiles (Figure 4A). This strategy employs a functionalized ruthenium complex and a fragment crystallizable (Fc) ligand anchored to a magnetic bead, enabling the localized generation of singlet oxygen near antibodies (Figure 4B). The short lifetime and limited diffusion of singlet oxygen ensure exclusive reactions with proximal histidine residues. This strategy maximizes the efficiency and specificity of functionalization, representing a significant advancement in the selective modification of antibodies.

This method demonstrated an innovative mode of selectivity, even though some drawbacks remain. For example, the 1-methyl-4-arylurazole used in these transformations is a strong electrophile that can react with Tyr, for instance, if such an amino acid is located near the reactive site. In addition, command of the DAR might have some limits, as the distance between the catalyst and the mAbs is not well defined, meaning that the photoredox reaction might not give satisfactory reproducibility.

#### Histidine/Tyr

**Figure 5:**
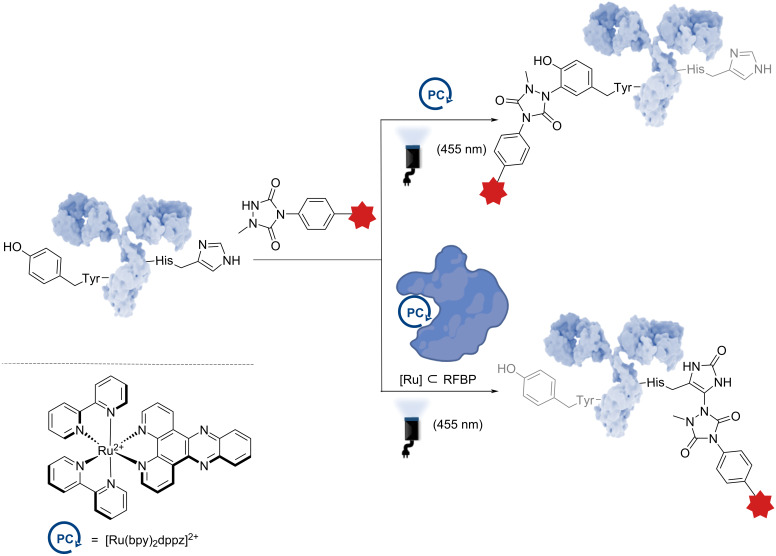
Schematic procedure of the divergent method developed by Sato et al. on histidine/tyrosine photoinduced modification. The antibody shown in this figure is from https://www.gettyimages.de/detail/illustration/monoclonal-antibody-igg2a-lizenfreie-illustration/585105259?searchscope=image%2Cfilm&adppopup=true; ALFRED PASIEKA/SCIENCE PHOTO LIBRARY, No. 585105259. This content is not subject to CC BY 4.0.

More recently, the same research group demonstrated the divergent functionalization of tyrosine or histidine, thanks to the use of a DNA photoswitch Ru complex, either used as a standalone system or involved in an artificial metalloenzyme (ArM). The use of the ArM significantly enhances the potential of the photocatalyst for antibody modification [43]. By inserting a [Ru(bpy)_2_dppz]^2+^ complex into the apo-form of riboflavin-binding protein (RFBP), a complete reversal of selectivity was achieved: the Ru complex alone enabled tyrosine modification via a photoredox reaction, whereas when embedded within the protein, the Ru-based ArM complex promoted histidine modification through an energy-transfer mechanism (Figure 5). Based on DFT calculations, this is permitted by the modification of the energy diagram of the ruthenium complex in the presence of biological and aqueous media. When applied to trastuzumab, the artificial metalloenzyme system ([Ru] ⊂ RFBP) thus yielded striking results – significantly reducing undesired tyrosine modifications on the heavy chain while enabling selective modification of histidine.

This impressive divergent method allows the same photocatalyst and electrophile to be involved in two different but selective bioconjugations of mAbs. It remains limited to reactions involving oxidative mechanistic pathways with ^1^O_2_.

#### Cys

**Figure 6:**
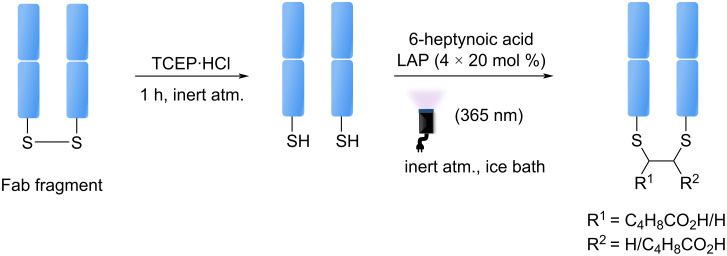
Schematic procedure developed by Bräse et al. on photoinduced disulfide rebridging method.

In 2016, Bräse et al. developed a photomediated disulfide rebridging method, exploiting the disulfide bridging sites in biomolecules to introduce specific functional groups (Figure 6) [44]. The bioconjugation reaction is based on thiol–yne coupling. Originally developed on peptides and proteins, this approach was applied to a fragment antigen-binding (Fab) antibody fragment (approximately 46 kDa) containing a single interchain disulfide bond. After 4 h of irradiation under UV wavelengths, the desired rebridged Fab fragment was obtained with around 40% conversion.

Although this method shows promise, it remains to be seen whether it is feasible for a full antibody, such as an IgG1, which typically contains four un-buried interchain disulfide bonds. Thus, rebridging allows the disulfide bonds to be reformed after conjugation, but one may question whether this could affect the long-term stability or functionality of the ADC, particularly if the new bonds do not perfectly mimic the properties of native disulfide bridges.

**Figure 7:**
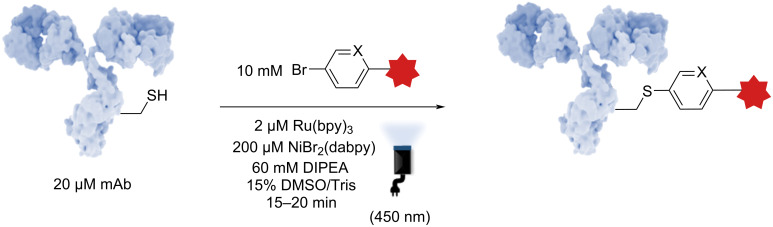
Schematic procedure developed by Lang et al. on a photoinduced dual nickel photoredox-catalyzed approach. The antibody shown in this figure is from https://www.gettyimages.de/detail/illustration/monoclonal-antibody-igg2a-lizenfreie-illustration/585105259?searchscope=image%2Cfilm&adppopup=true; ALFRED PASIEKA/SCIENCE PHOTO LIBRARY, No. 585105259. This content is not subject to CC BY 4.0.

More recently, Lang et al. developed an approach using dual photoredox/nickel-catalyzed antibody functionalization [45], demonstrating the selective modification of Cys residues through photocatalytic methods (Figure 7). A key advantage of this strategy lies in the use of readily available and inexpensive Ru(bpy)_3_, along with a water-soluble, air- and moisture-stable Ni(dabpy)Br_2_ catalyst. Moreover, they demonstrated that this system works efficiently in aqueous conditions, making it highly suitable for applications involving antibodies. These characteristics make the method highly attractive for industrial applications, where both scalability and robustness are essential.

Beyond its practical advantages, the method offers notable versatility, enabling the conjugation of a wide range of aryl linkers to Cys residues. This flexibility is crucial for the development of ADCs, where precise control over the linker and conjugation site is vital to optimizing therapeutic efficacy and pharmacokinetics. The Lang group's approach therefore represents a significant advancement in the field of selective and scalable antibody functionalization.

Importantly, although pioneering the field of dual nickel catalysis for the functionalization of antibodies in a very elegant and practical manner, the method has been applied to Cys, which necessitates the use of a reductant to access the free form of Cys. Another consequence was the high and non-reproducible DAR. The development of such methods applied to other canonical amino acids should benefit from the advantages of this approach while overcoming the encountered problems.

#### Glutamic acid

**Figure 8:**
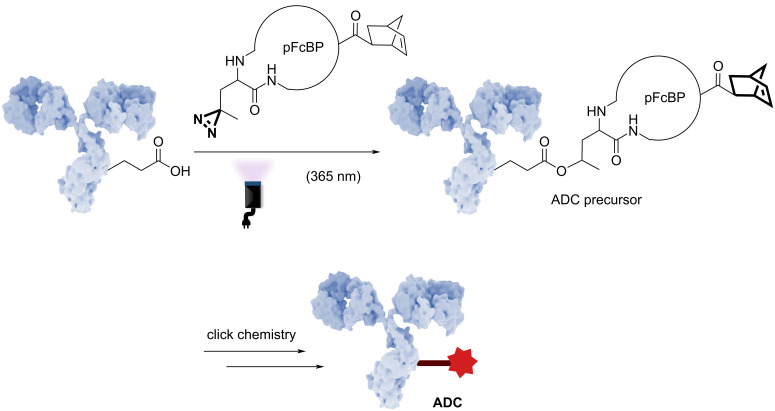
Schematic of the procedure developed by Chang et al. on photoinduced high affinity IgG Fc-binding strategy. The antibody shown in this figure is from https://www.gettyimages.de/detail/illustration/monoclonal-antibody-igg2a-lizenfreie-illustration/585105259?searchscope=image%2Cfilm&adppopup=true; ALFRED PASIEKA/SCIENCE PHOTO LIBRARY, No. 585105259. This content is not subject to CC BY 4.0.

The Chung group explored the development of a method based on a high affinity immunoglobulin G (IgG) Fc-binding strategy to generate ADCs (Figure 8). Although such methods are generally reported with a heterogeneous DAR, the group developed a site-specific approach leading to a homogeneous DAR 2, thanks to a photoreactive Fc-binding peptide derivative (pFcBP) containing a photoleucine (pLeu) [46]. By analyzing the X-ray crystal structure of IgG, the researchers identified high-affinity binding sites for the ligand within the Fc domain. This structural analysis allowed precise determination of amino acid residue positions and orientations.

Upon photoirradiation, the pLeu within the pFcBP generated a carbene from the diazirine moiety. This transformation facilitated a site-specific covalent linkage to the antibody, resulting in the formation of a DAR 2 ADC precursor. For their ADC design strategy, the group selected a polyethylene glycol (PEG) linker, as it enhances the water solubility of the ADC, while the payload consists of the cytotoxic agent DM1, which acts as a microtubule destabilizer. To ensure the stability and homogeneity of the final product, the design of the FcBP included a norbornene motif at the N-terminal end of the peptide sequence. The norbornene motif selectively reacts with a tetrazine located on the spacer-payload.

This study demonstrates the potency of photoinduced methods to access more homogeneous ADCs, hopefully reducing patient side effects.

In summary, photoredox modifications of antibodies have gained attention in recent years, though the field is still in its early stages because of the complexity of antibody structures. These photoinduced methods offer the potential for selective and mild functionalization, which could be particularly valuable for producing homogeneous ADCs with controlled properties. Even though very few photoredox approaches have been published in the literature when compared to proteins, highlighting the complexity of antibody architecture, new photoinduced methods for the selective modification of natural amino acids in antibodies are of high necessity.

### Discussion

**Figure 9:**
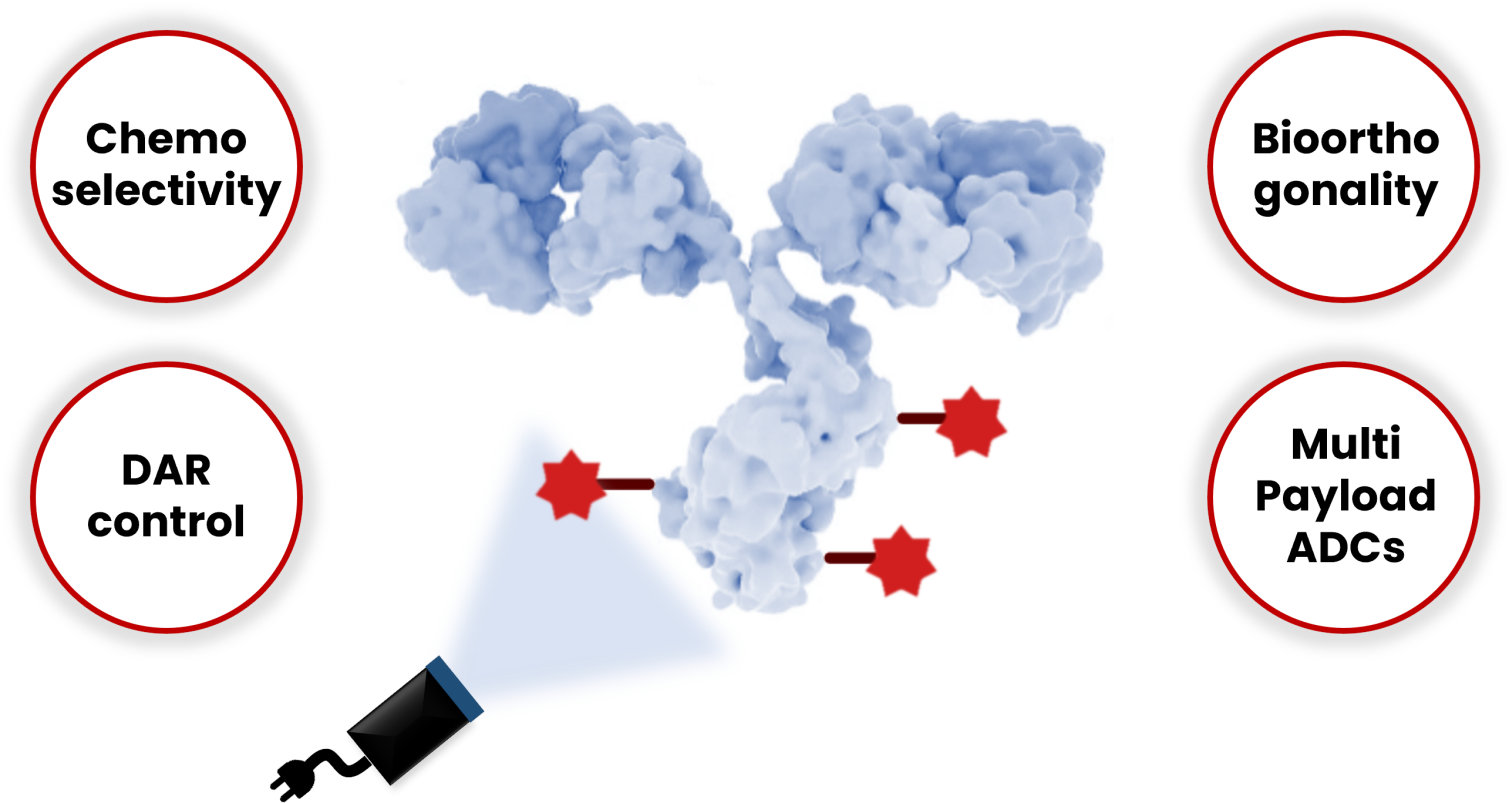
Potential advantages of photoredox chemistry for bioconjugation applied to antibodies. The antibody shown in this figure is from https://www.gettyimages.de/detail/illustration/monoclonal-antibody-igg2a-lizenfreie-illustration/585105259?searchscope=image%2Cfilm&adppopup=true; ALFRED PASIEKA/SCIENCE PHOTO LIBRARY, No. 585105259. This content is not subject to CC BY 4.0.

Tremendous inroads have already been made in the application of photochemical methods to the construction of ADCs. Highlighted below are additional aspects that photochemical transformations could, in our opinion, bring to ADCs that are not easily achieved through traditional, mostly two-electron mechanistic approaches (Figure 9).

#### Chemoselectivity

As mentioned in the introduction, there are exceedingly stringent requirements for the types of reactions that can be used for the elaboration of ADCs. Among the signature features of photochemical, single-electron transformations is their ability to be conducted in dilute aqueous media at room temperature and at neutral pH [47]. Additionally, the distinctive mechanistic paradigms under which photochemical reactions operate makes them uniquely tolerant of numerous unprotected functional groups [48]. Consequently, the elaboration of a variety of highly sophisticated biomolecules, including ADCs, can be carried out without protecting groups in a highly selective manner. This chemoselectivity should extend to any molecularly complex linkers and/or payloads being conjugated to the mAbs, allowing highly efficient entry to ADCs.

#### DAR control

**Figure 10:**
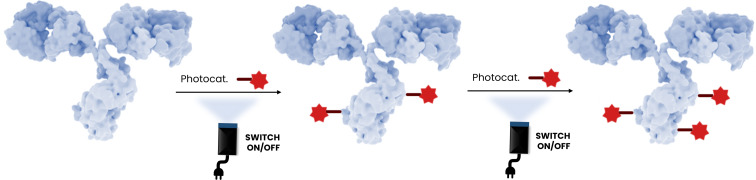
Representation of the photoinduced control of the DAR. The antibody shown in this figure is from https://www.gettyimages.de/detail/illustration/monoclonal-antibody-igg2a-lizenfreie-illustration/585105259?searchscope=image%2Cfilm&adppopup=true; ALFRED PASIEKA/SCIENCE PHOTO LIBRARY, No. 585105259. This content is not subject to CC BY 4.0.

A noteworthy characteristic of photochemical transformations is the ability to unambiguously start and stop reactions instantaneously using light on and light off reaction conditions (Figure 10). Aspirationally this would allow exquisite control of the drug/antibody ratio, taking advantage of the nature of photochemical transformations to occur rapidly and selectively at the kinetically most reactive sites [49–50]. Importantly, as a general comment, targeting a single class of amino acids, the most solvent-exposed would react preferentially to those buried within hydrophobic domains of the mAbs, and ideally the relative rates of their reactivity would translate directly to tightly controlled DARs. The time of irradiation, the wavelength of the light, the lamp wattage, and diverse photocatalysts or mechanisms (e.g., energy transfer, photoredox, or electron-donor/electron-acceptor photoinduced electron transfer) might all be brought to bear on controlling the DAR. In addition to the DAR, homogeneity for conjugation at specific sites using light control might also be improved using photochemical transformations. Importantly, optimization of bioconjugation reactions with technologies such as high-throughput experimentation has already been applied on antibodies [51].

#### Bioorthogonality

Bioorthogonal chemistry has transformed our capability to study and alter biological systems at the molecular level [52–53]. Owing to the often-unique mechanisms characterized by photocatalytically promoted reactions, reactivity patterns might be developed that allow selective reactions at amino acids that are not currently used for conjugation of linkers/payloads to the mAb. Potential access to modification of the mAbs at different amino acids has obvious ramifications for installation of the linker/payload because of the relative abundance and local environments of the amino acids, in addition to the inherent reactivity of these diverse components. In turn, this selectivity for reaction at a wider range of amino acids impacts several important features of ADCs, including site selection for the linker/payload, the DAR, and the homogeneity of the ADCs.

#### Multi-payload ADCs

**Figure 11:**
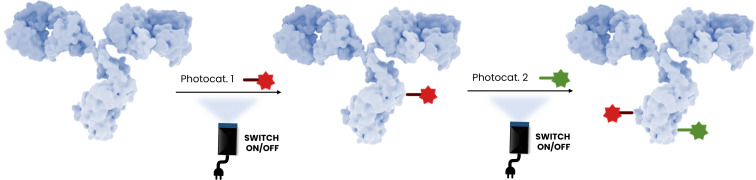
Representation of a photoinduced control of multi-payloads ADC strategy. The antibody shown in this figure is from https://www.gettyimages.de/detail/illustration/monoclonal-antibody-igg2a-lizenfreie-illustration/585105259?searchscope=image%2Cfilm&adppopup=true; ALFRED PASIEKA/SCIENCE PHOTO LIBRARY, No. 585105259. This content is not subject to CC BY 4.0.

Most clinically approved ADCs contain a single-drug payload. However, systemic cancer chemotherapies often involve combinations of drugs. Combination regimens improve treatment outcomes by producing synergistic anticancer effects and slowing the development of drug-resistant cell populations. Researchers aim to replicate combination regimens by developing techniques for attaching multiple payloads to a single antibody molecule with high homogeneity [54–56]. Generating homogeneous multi-payload ADCs is complex because of the diverse reactive functional groups on antibody surfaces. As outlined above in the discussion concerning bioorthogonality, it is conceivable that photochemical methods could be developed allowing multi-payload ADCs to be prepared effortlessly, as the single-electron mechanistic approach of such reactions is completely orthogonal to currently used methods, and even among photochemical methods that might be designed for different amino acids. The scheme below (Figure 11) represents schematically how bioorthogonal introduction of linkers/payloads on the ADCs might be carried out using photocatalyzed approaches to ADC construction. Importantly, native amino acids could be used in these processes, and thus the onerous and expensive introduction of non-canonical amino acids into the ADCs would be completely unnecessary. Photocatalyzed methods could also be used in conjunction with more traditional chemical approaches at either stage to allow biorthogonal strategies for multi-payloads ADCs.

#### Antibody–protein conjugates

Antibody–protein conjugates possess tremendous promise within the domains of biotechnology and biomedical research [57–58]. To date, such entities have most often been formulated from the expression of fusion proteins, although more recently post-translational protein–protein conjugation has been recognized as a means to access such structures [59]. However, methods developed to date based on traditional two-electron chemistry with canonical amino acids are extremely limited and are most effectively based on maleimide hydrolyzing methods that rely on a single amino acid (Cys) conjugation. The development of viable photochemical transformations targeted toward a variety of diverse canonical amino acids would introduce more versatility, efficiency, and convenience in the construction of these increasingly important structural units.

#### Cautionary considerations

Photoredox chemistry offers a powerful approach for the targeted modification of proteins and antibodies, enabling selective covalent bond formation and functionalization under mild conditions. However, its use in biomolecule modification comes with several specific features that require consideration. Some of these are detailed below.

**Photoaggregation:** The possibility of photoinduced aggregation during photoconjugation reactions represents a major challenge for the stability and efficacy of conjugated antibodies [60]. Exposure of antibodies to light, particularly UV light, can trigger photoinduced reactions that compromise their structural stability. These phenomena include the oxidation of sensitive residues such as tryptophan (Trp), tyrosine (Tyr), cysteine (Cys), and histidine (His), as well as partial unfolding of the antibodies, exposing hydrophobic regions that promote nonspecific interactions and aggregate formation. Additionally, covalent cross-links may form, involving disulfide bridges or Cys–Tyr bonds, further increasing the risk of aggregation.

Photosensitive payloads, although essential for specific therapeutic or diagnostic applications, are particularly vulnerable to these photochemical reactions. Their light-induced modifications not only exacerbate aggregation but may also reduce therapeutic efficacy and increase the risk of immunogenicity. These effects are especially concerning when the DAR is high, as a higher DAR increases the overall photosensitivity of the conjugated antibody.

Therefore, it is crucial to account for these factors during photoconjugation reactions to mitigate aggregation phenomena. Strategies such as optimizing the length and nature of the linker, precisely controlling reaction conditions (light intensity, exposure time), or modifying conjugation sites to distance payloads from sensitive residues may offer effective solutions. Finally, storage and handling of conjugated antibodies require special attention to minimize exposure to ambient light, which could contribute to structural instability and promote aggregation over time.

**Reaction time:** Proteins and antibodies are delicate macromolecules, and prolonged exposure to light or reactive intermediates can lead to protein denaturation or aggregation [61]. Because photoredox reactions often generate highly reactive species (such as radicals or singlet oxygen), long exposure times could cause unwanted side reactions, including the degradation of 3D structure of the protein or antibody. A short reaction time minimizes this risk by allowing the reaction to proceed quickly and efficiently before damage occurs. To avoid damage caused by high energy wavelength, the use of irradiation in the red region could be considered [62–65].

**Aqueous media:** Developing photoredox reactions compatible with aqueous media is essential for the modification of antibodies, as these biomolecules are typically dissolved in water or aqueous buffers to maintain their stability and activity. Antibodies are sensitive to their environment, and using a non-negligible amount of organic solvents or harsh chemical conditions can lead to denaturation, aggregation, or loss of functionality. Buffers, which are commonly used to stabilize antibodies by maintaining their pH and ionic strength, must also be compatible with the photoredox process to prevent unwanted side reactions or instability.

Thus, although photoredox chemistry offers a promising method for the targeted modification of antibodies, careful consideration of factors such as photoaggregation, reaction time, and aqueous media compatibility is essential to ensure the stability, efficacy, and functionality of the conjugated biomolecules.

## Conclusion

In conclusion, the development of antibody–drug conjugates (ADCs) holds great promise for advancing targeted therapeutics, particularly in the treatment of cancer and other refractory diseases. However, the challenges related to achieving selective, efficient, and stable conjugation of antibodies to their payloads remain a significant barrier to realizing the full potential of ADCs. Traditional conjugation methods often struggle with issues such as site-selectivity and heterogeneous products. Photoredox chemistry has emerged as a transformative approach, offering precise control over the modification of antibodies with the potential to enhance ADC purity, stability, and therapeutic efficacy. By utilizing light-driven chemical transformations, photoredox techniques enable selective functionalization of antibodies at specific sites, addressing the limitations of conventional methods. As research in photochemistry continues to evolve, these strategies may pave the way for the creation of more homogeneous and optimized ADCs, enhancing their therapeutic outcomes and minimizing off-target effects. This perspective highlights the exciting future of photoredox methods in the development of next-generation ADCs, ultimately contributing to the broader landscape of targeted biomedicine.

## Data Availability

All data that supports the findings of this study is available in the published article and/or the supporting information of this article.

## References

[1] Fu Z, Li S, Han S, Shi C, Zhang Y (2022). Signal Transduction Targeted Ther.

[2] Qian L, Lin X, Gao X, Khan R U, Liao J-Y, Du S, Ge J, Zeng S, Yao S Q (2023). Chem Rev.

[3] Buttgereit F, Aelion J, Rojkovich B, Zubrzycka-Sienkiewicz A, Chen S, Yang Y, Arikan D, D'Cunha R, Pang Y, Kupper H (2023). Arthritis Rheumatol.

[4] Samantasinghar A, Sunildutt N P, Ahmed F, Soomro A M, Salih A R C, Parihar P, Memon F H, Kim K H, Choi K H (2023). Biomed Pharmacother.

[5] Hobson A D, Xu J, Marvin C C, McPherson M J, Hollmann M, Gattner M, Dzeyk K, Fettis M M, Bischoff A K, Wang L (2023). J Med Chem.

[6] Usama S M, Thapaliya E R, Luciano M P, Schnermann M J (2021). Curr Opin Chem Biol.

[7] Lin D, Lechermann L M, Huestis M P, Marik J, Sap J B I (2024). Angew Chem, Int Ed.

[8] Wu K-L, Yu C, Lee C, Zuo C, Ball Z T, Xiao H (2021). Bioconjugate Chem.

[9] Park J, Lee S, Kim Y, Yoo T H (2021). Bioorg Med Chem.

[10] von Witting E, Hober S, Kanje S (2021). Bioconjugate Chem.

[11] Walsh S J, Bargh J D, Dannheim F M, Hanby A R, Seki H, Counsell A J, Ou X, Fowler E, Ashman N, Takada Y (2021). Chem Soc Rev.

[12] Coumans R G E, Ariaans G J A, Spijker H J, Renart Verkerk P, Beusker P H, Kokke B P A, Schouten J, Blomenröhr M, van der Lee M M C, Groothuis P G (2020). Bioconjugate Chem.

[13] Garbaccio R M, Knochel P (2014). Chemistry of Antibody–Small Molecule Drug Conjugates. Comprehensive Organic Synthesis.

[14] Sochaj A M, Świderska K W, Otlewski J (2015). Biotechnol Adv.

[15] Kang M S, Kong T W S, Khoo J Y X, Loh T-P (2021). Chem Sci.

[16] Lu J, Jiang F, Lu A, Zhang G (2016). Int J Mol Sci.

[17] Porte K, Riberaud M, Châtre R, Audisio D, Papot S, Taran F (2021). ChemBioChem.

[18] Lemieux G A, Bertozzi C R (1998). Trends Biotechnol.

[19] Hackenberger C P R, Schwarzer D (2008). Angew Chem, Int Ed.

[20] Stump B (2022). ChemBioChem.

[21] Haque M, Forte N, Baker J R (2021). Chem Commun.

[22] McCombs J R, Owen S C (2015). AAPS J.

[23] Jain N, Smith S W, Ghone S, Tomczuk B (2015). Pharm Res.

[24] Yuan D, Zhang Y, Lim K H, Leung S K P, Yang X, Liang Y, Lau W C Y, Chow K T, Xia J (2022). J Am Chem Soc.

[25] Natesan R, Dykstra A B, Banerjee A, Agrawal N J (2023). Antibiotics (Basel, Switz).

[26] Alley S C, Benjamin D R, Jeffrey S C, Okeley N M, Meyer D L, Sanderson R J, Senter P D (2008). Bioconjugate Chem.

[27] Shen B-Q, Xu K, Liu L, Raab H, Bhakta S, Kenrick M, Parsons-Reponte K L, Tien J, Yu S-F, Mai E (2012). Nat Biotechnol.

[28] Maruyama K, Ishiyama T, Seki Y, Sakai K, Togo T, Oisaki K, Kanai M (2021). J Am Chem Soc.

[29] Xie X, Moon P J, Crossley S W M, Bischoff A J, He D, Li G, Dao N, Gonzalez-Valero A, Reeves A G, McKenna J M (2024). Nature.

[30] Zhang Y, Tan J, Chen Y (2023). Chem Commun.

[31] Holland J P, Gut M, Klingler S, Fay R, Guillou A (2020). Chem – Eur J.

[32] Lechner V M, Nappi M, Deneny P J, Folliet S, Chu J C K, Gaunt M J (2022). Chem Rev.

[33] Aguilar Troyano F J, Merkens K, Anwar K, Gómez‐Suárez A (2021). Angew Chem, Int Ed.

[34] Kostova V, Désos P, Starck J-B, Kotschy A (2021). Pharmaceuticals.

[35] Hobson A D (2024). RSC Med Chem.

[36] Dudchak R, Podolak M, Holota S, Szewczyk-Roszczenko O, Roszczenko P, Bielawska A, Lesyk R, Bielawski K (2024). Bioorg Chem.

[37] Ryu K A, Kaszuba C M, Bissonnette N B, Oslund R C, Fadeyi O O (2021). Nat Rev Chem.

[38] Li P, Terrett J A, Zbieg J R (2020). ACS Med Chem Lett.

[39] Liu J-Q, Shatskiy A, Matsuura B S, Kärkäs M D (2019). Synthesis.

[40] Bottecchia C, Noël T (2019). Chem – Eur J.

[41] Liu Z, Okamoto Y, Sato S (2024). ChemCatChem.

[42] Nakane K, Sato S, Niwa T, Tsushima M, Tomoshige S, Taguchi H, Ishikawa M, Nakamura H (2021). J Am Chem Soc.

[43] Okamoto Y, Mabuchi T, Nakane K, Ueno A, Sato S (2023). ACS Catal.

[44] Griebenow N, Dilmaç A M, Greven S, Bräse S (2016). Bioconjugate Chem.

[45] Bacauanu V, Merz Z N, Hua Z L, Lang S B (2023). J Am Chem Soc.

[46] Lee T, Kim J H, Kwon S J, Park S H, Kim J, Kang H J, Chung S J (2020). Org Lett.

[47] Russo C, Brunelli F, Tron G C, Giustiniano M (2023). J Org Chem.

[48] Shaw M H, Twilton J, MacMillan D W C (2016). J Org Chem.

[49] Tower S J, Hetcher W J, Myers T E, Kuehl N J, Taylor M T (2020). J Am Chem Soc.

[50] Kim J, Li B X, Huang R Y-C, Qiao J X, Ewing W R, MacMillan D W C (2020). J Am Chem Soc.

[51] Emmert M H, Yang C, Kwan E E, Chmielowski R, Kilgore B, VanAernum Z L, Bottecchia C, Barrientos R C, Haley M, Raymond K (2025). Org Process Res Dev.

[52] Kim J, Xu Y, Lim J H, Lee J Y, Li M, Fox J M, Vendrell M, Kim J S (2025). J Am Chem Soc.

[53] Mato M, Fernández-González X, D'Avino C, Tomás-Gamasa M, Mascareñas J L (2024). Angew Chem, Int Ed.

[54] Journeaux T, Bernardes G J L (2024). Nat Chem.

[55] Tsuchikama K, Anami Y, Ha S Y Y, Yamazaki C M (2024). Nat Rev Clin Oncol.

[56] Journeaux T, Geeson M B, Murray T V, Papworth M A, Gothard M, Kettle J G, Vasco A V, Bernardes G J L (2025). Angew Chem, Int Ed.

[57] Bell M R, Engleka M J, Malik A, Strickler J E (2013). Protein Sci.

[58] Mahmood T, Shahbaz A, Hussain N, Ali R, Bashir H, Rizwan K (2023). Int J Biol Macromol.

[59] Vasco A V, Taylor R J, Méndez Y, Bernardes G J L (2024). J Am Chem Soc.

[60] Cockrell G M, Wolfe M S, Wolfe J L, Schöneich C (2015). Mol Pharmaceutics.

[61] Pattison D I, Davies M J (2006). Actions of ultraviolet light on cellular structures. Cancer: Cell Structures, Carcinogens and Genomic Instability.

[62] Sellet N, Cormier M, Goddard J-P (2021). Org Chem Front.

[63] Connell T U (2022). Dalton Trans.

[64] Linsley C S, Quach V Y, Agrawal G, Hartnett E, Wu B M (2015). Drug Delivery Transl Res.

[65] Ravetz B D, Tay N E S, Joe C L, Sezen-Edmonds M, Schmidt M A, Tan Y, Janey J M, Eastgate M D, Rovis T (2020). ACS Cent Sci.

